# Test Groups, Not Individuals: A Review of the Pooling Approaches for SARS-CoV-2 Diagnosis

**DOI:** 10.3390/diagnostics11010068

**Published:** 2021-01-04

**Authors:** Renato Millioni, Cinzia Mortarino

**Affiliations:** 1 Fascial Manipulation Institute by Stecco, 35129 Padova, Italy; 2 Department of Statistical Sciences, University of Padova, 35121 Padova, Italy; cinzia.mortarino@unipd.it

**Keywords:** pooling, molecular testing, SARS-CoV-2

## Abstract

Massive molecular testing for SARS-CoV-2 diagnosis is mandatory to manage the spread of COVID-19. Diagnostic screening should be performed at a mass scale, extended to the asymptomatic population, and repeated over time. An accurate diagnostic pipeline for SARS-CoV-2 that could massively increase the laboratory efficiency, while being sustainable in terms of time and costs, should be based on a pooling strategy. In the past few months, researchers from different disciplines had this same idea: test groups, not individuals. This critical review intends to highlight both the general consents—even if the results from different publications have been obtained with different protocols—and the points of disagreement that are creating some interpretative/comprehension difficulties. Different pooling schemes and technical aspects associated to the type of pooling adopted are described and discussed. We hope that this review can consolidate information to support researchers in designing optimized COVID-19 testing protocols based on pooling.

## 1. Introduction

The outbreak of SARS-CoV-2 in December 2019 in China quickly became a pandemic emergency. Until vaccines are widely available, prevention is based on case isolation, contact tracing, quarantine, physical distancing, and hygiene measures. To minimize the viral contagion, increasing our ability to monitor SARS-CoV-2 spread in the general population is mandatory. Diagnostic screening should be performed at a mass scale that could be statistically significant, extended to the asymptomatic population, and repeated over time. No serological test is able to detect the presence of the virus in the early stages of infection, in which the subject is contagious even in the absence of symptoms [1]. Therefore, nucleic acid tests offer the earliest and most sensitive detection for the presence of SARS-CoV-2. The standard protocol for the Covid-19 diagnosis is performed by RT-PCR on upper respiratory specimen, e.g., nasopharyngeal (NP) swabs. Recent studies have shown that the same protocol can also be correctly applied on saliva, with the advantage of making sample collection easier [2]. Nevertheless, as monitoring capacity is limited, testing in most countries is generally focused on ill patients, while asymptomatic, yet potentially infectious carriers remain undiagnosed. Moreover, the number of subjects tested for SARS-CoV-2 varies significantly by country, being lower in developing countries. There are several reasons for this, including lack of laboratory facilities and reagents [3].

Thus, the challenge is: how can we devise a diagnostic pipeline for SARS-CoV-2 that is both accurate and sustainable? A pandemic emergency can hardly be managed with standard approaches. A possible proposed solution to massively scaling up COVID-19 testing by orders of magnitudes is sample pooling. The change of perspective is striking: increase diagnoses by doing fewer tests. In the past few months, researchers from different disciplines had this same idea and rapidly mobilized to propose original solutions. Sample pooling can increase testing, with substantial reductions in terms of time, costs, and need for chemical reagents. Now, the following question has arisen: why is such a promising and convenient proposal not universally adopted by clinical laboratories or why this is happening so slowly?

First, for clinical biochemist wont to applying individualized protocols, pooling may be seen as a source of analytical error. The caution towards an unorthodox protocol could partly explain the slow adoption of pooling by clinical labs. We think there are also some difficulties in reaching clear and unambiguous information on this proposal. The abovementioned publications, although based on the idea of pooling, differ in both a theoretical and a practical point of view. Although sample pooling is an appealing strategy to minimize the number of tests, it is difficult to extricate the most feasible one among the different possibilities to perform this method. Moreover, a question remains: how do analytical errors affect this procedure?

This critical review intends to highlight both the general consents—even if the results have been obtained with different protocols—and the critical points of disagreement that are creating some interpretative/comprehension difficulties.

We hope that this review can urgently consolidate information to support researchers in designing optimized COVID-19 testing protocols based on pooling.

## 2. Pooling: The Proof of Principle

In binary high-throughput screening projects, where the aim is to identify low-frequency events, pooling is a natural solution: it lessens the number of tests, minimizing the cost of the single assay in terms of time and money.

Typically, a successful pooling strategy requires three conditions:

1. Only a pool made up of all negative samples will give a negative result for the pool analysis;
2. A single positive sample within a pool makes the result of the pool analysis positive. If the pool is positive, it is necessary to proceed to further testing, to identify the true positives (TP);
3. Pooling is especially efficient when the fraction of expected positives is small. In fact, as all individual samples in a negative pool are considered as true negative (TN), the pooling approach significantly reduces time and cost when a large proportion of pools tests as negative.

While the first two points are clear, the third has been so far, in our opinion, a source of misunderstanding. The take home message is that the pooling method is effective only for low virus frequencies. Unfortunately, little clarity has been made about how low is “low”: some studies set this boundary at 1% or less [4], others go as high as 5% [5] or 10% [6,7]. From a mathematical point of view, the low frequency limit rises further, as some pooling strategies are significantly effective even for frequencies up to 20%. This means that there is no reason to segregate pooling strategies for the monitoring of contagions at the end of the pandemic, when very low viral frequencies are expected, such as about 1: 1000. The advantages of the pooling approach would be significant even during the peak contagion phase.

## 3. Pooling Scheme: The Theoretical Point of View

The articles on pooling focus on either one or both of these aspects: (i) which is the most appropriate pooling scheme to adopt and (ii) how to actually create these pools in the laboratory.

**Figure 1:**
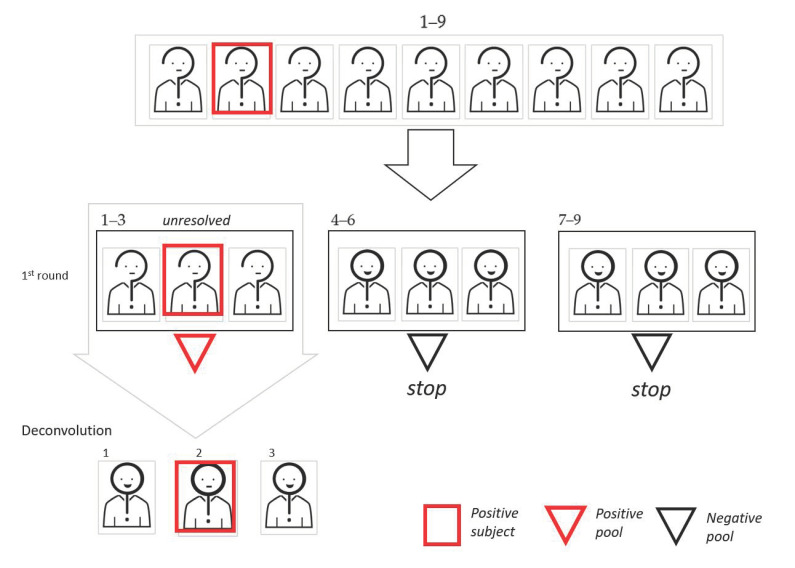
Graphic representation of the mono-dimensional (1D) pooling approach. In this example, the cohort dimension is 9, the pool size is 3, and the virus frequency, *vf,* is 0.10. To begin, aliquots of the nine samples are used to create three pools. Since two pools result as negative, we can exclude six subjects. The remaining three subjects of the pool that tested as positive are retested individually.

Starting with the pooling scheme, the simplest way to pool is a mono-dimensional (1D) protocol, where a number of samples are mixed before performing the experimental workup; then, only individual samples of the pools that gave a positive result are re-tested (deconvolution process). In Figure 1, we show a graphic representation of the 1D pooling scheme.

The roots of this idea go back to Dorfman [8]. In the past few months, multiple groups proposed to apply this approach to SARS-CoV-2 detection. One at Stanford [9], in particular, was the first to publish the results of pooling for SARS-CoV-2 detection, with the merit of having also verified the idea’s feasibility on biological samples. The described approach is the simplest one and it is characterized by a single pooling step, 1D, after which further individual tests may be required. Since this March, dozens of preprints of academic papers on this topic have become available with statistical simulations, mathematical formulas, and algorithmic models to design the pooling procedure.

Alternative approaches refine the 1D procedure by introducing further pooling steps. Now we are almost spoiled for choice, but even if there are many ideas, we can classify most of the multiple pooling steps schemes as belonging to two macro categories: adaptative and non-adaptative.

**Figure 2:**
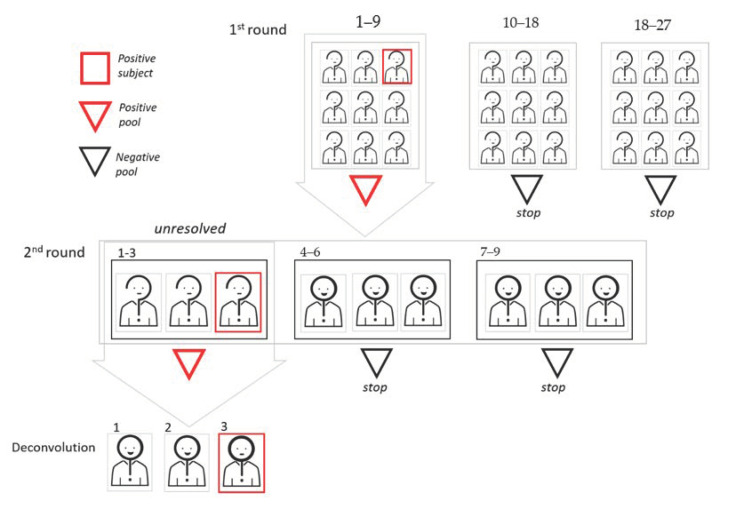
Graphic representation of the two-dimensional (2D) pooling approach. In this example, the cohort dimension is 27, and the first round pool size is 9. Since two pools result as negative in the first round, 12 subjects can be excluded. Sample aliquots from the nine subjects of the pool that tested as positive are used for a 1D pooling approach, where the pool size is 3.

In the adaptative methods, the results from the earlier pools tests determine who is selected to build the subsequent pooling steps. Subjects belonging to negative pools are excluded from further investigations, while positive pools require further steps by analyzing its components. If the number of positive pools is large, the most frequent choice is to proceed with the creation and testing of second-level pools, with subsequent individual analysis only of the samples belonging to the positive second-level pools [9,10,11,12]. In the end, individual tests for sample unresolved by pooling have to be performed (deconvolution). Second-level pools can be built either by splitting positive pools in subpools (thus reducing the pool size at the second step) or rearranging samples completely (to use the same pool size in the two steps). Different strategies to build second-level subpools from positive first-level pools are discussed in [13]. Notice that, due to the structure of adaptative schemes, the different pooling steps have to be performed sequentially (in order to build the second pooling step, results of the first steps have to be known). This approach may slow down the analysis, but has the advantage of consistently reducing the tests to be performed in steps after the first one. In Figure 2, we show a graphic representation of two-dimensional (2D) pooling, in which positive first-level pools are split in smaller second-level subpools.

**Figure 3:**
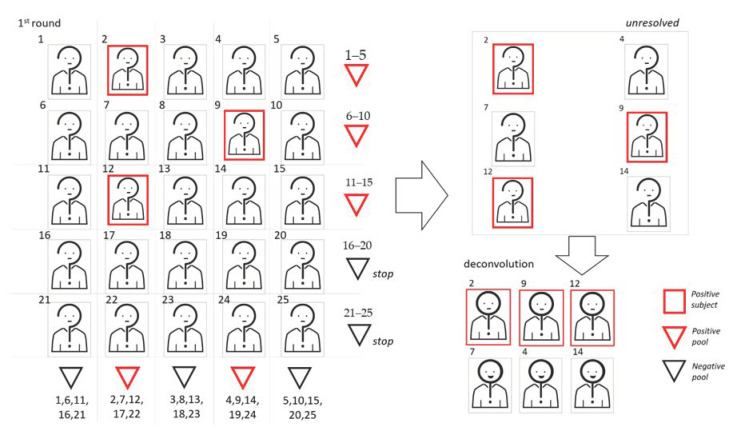
We show a graphic representation of 2D pooling, where samples are arranged on the basis of a symmetric matrix. In this example, the cohort dimension is 25 and the pool size is 5. To begin, samples are used to create 10 pools, 5 following the rows and 5 following the column of the matrix. Samples belonging to at least two positive pools will be retested individually.

In Figure 3, we show a graphic representation of 2D pooling, where subjects in positive pools are rearranged for the second pooling step.

**Figure 4:**
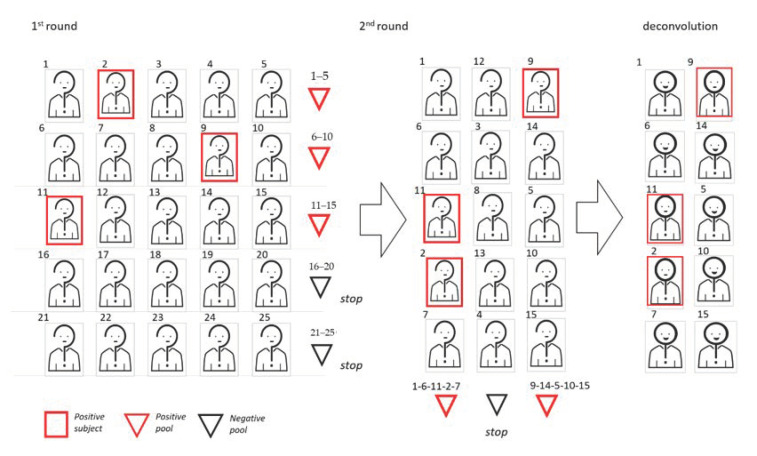
Graphic representation of the 2D sequential pooling approach. In this example, the cohort dimension is 25 and the pool size is 5. To begin, samples are arranged on a matrix and used to create five pools following the rows. Among these first-round pools, two turn out to be negative. The 15 remaining subjects are then rearranged in 3 vertical pools. Since two pools give a positive result, 10 validation tests are required.

In Figure 4, we show a graphic representation of the 2D pooling with the sequential approach.

**Figure 5:**
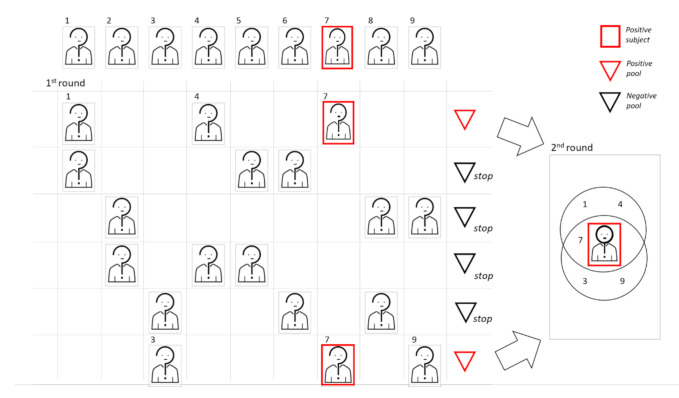
Graphic representation of the combinatorial pooling approach. In this example, two replicates of each sample are created and multiple pool with a size of 3 are tested simultaneously. Samples belonging to at least two positive pools are regarded as positive.

In non-adaptative approaches, replicates of each sample are created (from 2 to 6 replicates) and multiple pool steps are performed simultaneously (making the results available earlier than for adaptative schemes) [4,5,14,15]. Combinatorial techniques are used to avoid that more than one replicate of the same sample occurs in the same pool. Identification of positive samples simultaneously uses all the results: samples with at least one replicate in a negative pool are considered as negative. Other samples may be unresolved (when pool results are not sufficient to establish whether a sample is positive or negative). Therefore, the combinatorial scheme should be designed in a such way to limit as much as possible the number of pools in which any pair of samples can co-occur: this is essential to reduce the number of unresolved samples and to increase the probability to identify the TP. The strength of this approach is that the number of unresolved samples is very small, especially when the virus frequency is low. At the same time, higher frequencies may reduce the advantage of combinatorial schemes (due to the increased probability that any replicate occurs with a positive sample). Shental et al. [4] stated that their method can correctly identify positive samples in the tested population with a viral frequency of up to 1.3%. In Figure 5, we show a graphic representation of the combinatorial pooling.

In adaptative approaches, when a pool is positive, each sample within the pool may need to be individually tested. Therefore, the volume of samples initially collected from an individual must be enough for both the pooled testing and individual follow-up testing. For non-adaptative approaches, appropriate procedures have to be used to create the desired number of replicates. Getting multiple replicates can be a serious problem because a greater quantity of material is needed, and if the starting sample contains a low viral quantity, the risk of false negatives could increase. We discuss this issue in the next paragraph. Notice that individual tests may prove to also be necessary in non-adaptative schemes, for unresolved samples.

A comparative evaluation of the different methods might prove challenging. Luckily, it is probably also superfluous. The studies about pooling that have been published so far tackle the problem by different approaches, always coming to a solution that gives interesting insights. In principle, it may be more convenient to focus on the shared ground, trying to understand if it is possible to extrapolate practical indications to structure an experimental design based on pooling.

The first consensus shared by the various studies is that the problem to be solved involves three parameters: *N*, which is the number of samples to be analyzed, *vf*, which represents the viral frequency (the prevalence of the virus in the population under study), and *s*, which is the size of the pool, i.e., the number of samples combined into a single batch. The efficiency of a pooling strategy, that is, the savings in terms of analyzed swabs as compared to single swab processing, depends on the combination of *N*, *vf*, and *s*. The *vf* obviously cannot be decided and is unknown; however, in practice, it should be estimated and updated on a daily basis, for each lab, and for a given geographic area or cohesive groups such as health care workers. Given an estimate for *vf*, *s* should be calibrated, because large pools are very efficient with low *vf* (many pools give a negative outcome), while higher *vf* should be dealt with smaller pools. Most studies agree that for *vf* values around 1%, an optimal pool size *s* is around 20–30. The optimal pool size decreases with the increase of *vf*. The *s* value drops to around 15 per *vf* of around 5%. For even higher frequencies (10–20%) the most suitable *s* value ranges between 4 and 8. [5,6,11,12]. According to other models, the optimal pool size could be even smaller, with a size of 11, 5, 4, and 3 for, respectively, a *vf* of 1, 5, 10, and 30% [16].

However, studies in which pools have a size of 64, 120 [5,16], and even 1000 [12,17] have also been done. These studies are useful for technical aspects; however, in practice, the models and statistical simulations currently available clearly suggest to use smaller pools. This is an advantage because technical difficulties clearly limit the maximal pool size and larger pools may increase the number of false negatives as we will explain later.

Verwilt et al. [7], relying on a simulation study (with five simulations for each configuration), found an inverse relationship between the efficiency gain of pooling and the viral prevalence over a range from 0.01% to 10%, but also observed that the comparison of efficiency between a two-dimensional pooling and a simple pooling approach depends on the prevalence value (none of the methods is uniformly superior to the others). For very small prevalence, i.e., below 0.4%, a single pooling step with high pool size is the most efficient. For higher prevalence values, appropriate two-step schemes provide better values. In another study [6], a simple 1D pooling strategy is compared to a 2D procedure at different *vf* and *s* values. In this comparison, 5000 simulations for each setting show that when very small pools are adopted (*s* = 5), different methods give equivalent results. As *s* increases from 8 to 20, the 2D pooling shows a better performance up to *vf* = 15%. For bigger pools (*s* = 24, 30), the same result with a *vf* value around 10% is observed.

**Figure 6:**
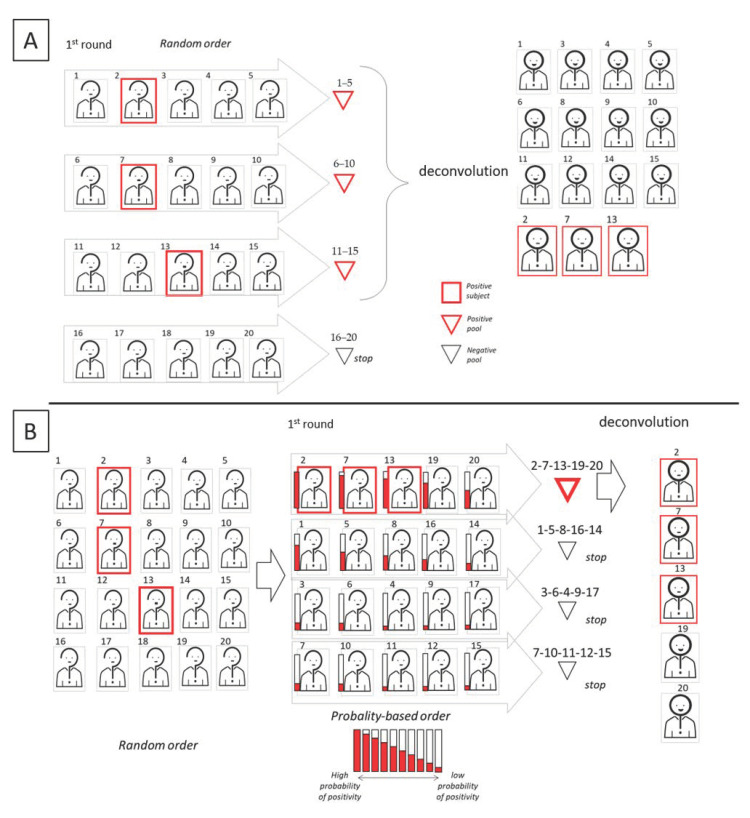
Graphic representation of the informed sequential pooling approach. In this example, the cohort dimension is 20, the pool size is 5. Upper (**A**) and lower (**B**) panels show two possible scenarios based on available information allowing for the classification of subjects as either “suspect positive” or “suspect negative.” In scenario B, information allows for a concentration of all the positive subjects in the same pool and so improve the efficiency of the pooling scheme.

The standard diagnostic protocol has so far been applied in an emergency situation, where priority was given mostly to those who manifested symptoms. However, in this context, independence among tests is assumed, while other possible correlations are neglected. Similarly, pools are also created randomly. However, the effects of a non-random pool creation have been studied for the first time by Millioni et al. [6]. In the “informed” pool creation, a score about the probability to be infected is associated to each subject, in order to tag the subject as suspected positive or negative. The correct assignment of this score should be accomplished by compiling a dedicated questionnaire where the score is calculated on the basis of clinical and epidemiological criteria that have already been associated to COVID-19. The aim is to cluster all the subjects with higher probability to be positive in the same pools, avoiding their random spreading in different pools. Additionally, family members or co-workers should preferably be pooled together. This type of grouping can greatly improve the efficiency of any pooling scheme. Similarly, Escobar et al. [18] recently developed a machine learning method that uses clinical and sociodemographic data from patients to order samples according to the predicted probability of yielding a positive result in the test, thus increasing the efficiency of pooled molecular testing. In Figure 6, we show a graphic comparison of pooling (upper panel) vs informed pooling (lower panel).

Evaluation of efficiency gain is performed differently in the analyzed research studies. Many of them discuss thoroughly the sensitivity of pools, but report efficiency data only about a single clinical application [9,10,19,20,21,22,]. Other papers evaluate, at least approximately, the probability of obtaining a negative pool or the expected number of tests required, as a function of *N*, *vf*, and pool size, *s* [11,12,15,16,22]. Only a few studies focus on variability of the number of required tests due to the random assignment of samples to pools around expected value; lucky assignments concentrate positive samples in a small number of pools, generating efficiency values much better than the expected value. On the contrary, we may also encounter assignments with higher spread of positive samples in the pools, generating efficiency values far from the expected value. Papers examining this topic, which could be very important in practical applications, rely on simulations to assess such a discrepancy [6,7]. Finally, we notice that the number of required tests in non-adaptative combinatorial methods is fixed and known, since it depends on the pooling scheme. For this approach, assignment of samples to pools affects the number of unresolved samples, whose expected value has to be evaluated [4,5,14,15].

## 4. Type of Pooling: Technical Laboratory Aspects

Testing for SARS-CoV-2 begins with the collection of a patient swab sample which is stored and transported in a viral transport medium (VTM) that maintains viral viability for 48 h at room temperature. These samples are then lysed and RNA is typically purified using kits based on either RNA extraction columns or magnetic beads. RNA extraction contains viral as well as human RNA; the latter is used for control. The detection of selected nucleic acid sequences is then performed by quantitative RT-PCR.

Currently, three different types of pooling have been proposed: (1) the swab pooling, which is obtained by adding swabs from multiple patients into a single volume of transport media, (2) the VTM pooling, which is obtained by collecting aliquots of transport media from different samples to create a homogeneous pool, and (3) the nucleic acid pool (NA pool), where an aliquot of nucleic acid extracted from each sample is collected to create a homogeneous pool.

Pooling before RNA extraction, as in the swab and in the VTM pooling, is the most convenient solution to save time and reagents, but it hinders to flag poorly taken samples in which there is no RNA or where the human RNA control is missing.

Swab pooling has been proposed by Schmidt et al. [23] with an alternative protocol based on two phases: (*i*) five minutes of incubation under constant agitation of a respiratory swab in a single reference tube with 4.3 mL of guanidinium thiocyanate buffer, and (*ii*) transfer and five minutes of incubation under constant agitation of the swab in a mini-pool tube with 2 mL of guanidinium thiocyanate buffer. These mini-pools can contain up to 10 samples each and, in the case of a positive pool result, deconvolution testing is carried out using the samples from the reference tubes.

It remains undetermined how many specimens are left behind in the reference tube and how many could be collected for the pool tube. As the extraction for the pooled tube takes place later and in a smaller volume, it could be assumed that most of the starting sample amount will remain in the reference tube, reducing the sensitivity of the pool analysis. Despite this, the authors found no sensitivity problems. This assumption suggests that it could be useful to investigate how much of the material collected by the swab elutes in the VTM solution and how much is left behind in the cotton, even in the standard protocol.

If the swab pool allows to test up to 10 samples simultaneously, most studies that use the VTM pool type are characterized by a larger size, with *s* between 30 and 50. However, the volume of media that can be used to create the pool depends on the loading capacity of the extraction protocol. For example, if the capacity of the extraction system is 1 mL, the creation of a 10-specimen pool size will require the withdrawal of 0.1 mL from each sample. In this way, if the pool contains a single positive sample, the creation of the pool causes a 10-fold dilution of the target viral sequences. This dilution will be even greater for larger pools. In particular, the non-adaptative pooling schemes that require the creation of multiple pools, in which a sample must be split into at least three or six replicates, worsen the risk of increasing false negatives because the starting quantity of the RNA in the NP buffer is not only diluted in pools but also fractionated upstream.

As a matter of fact, after RNA extraction, samples are collected in smaller volumes; hence, the problem of dilution for NA pool is lessened, but still possible. Using this type of pooling, the size of the pool further increases, with up to 1000 samples analyzed simultaneously [17].

As mentioned above, the models and statistical simulations currently available clearly suggest that the creation of huge pools (100–1000 subjects) is not necessary, independently of the pooling scheme followed. This is an advantage, since the pool size should be kept as low as possible to reduce the dilution effect and, at the same time, maintaining the sensitivity of the assay and increasing the process efficiency. Moreover, as the size of the pool increases, the volume to be taken from each sample decreases, which is associated with pipetting errors, especially if the laboratory does not have automated systems for these operations. Some studies, in which very small pools (*s* = 5) were used, reported no dilution errors [23,24]. Conversely, an increase of about 10% of false negatives was observed when larger pools (>30) were adopted [17,25].

The number of samples that can be pooled without affecting the PCR sensitivity is limited by the PCR cycle threshold (Ct) for the target, i.e., the cycle at which amplification becomes detectable over background noise. Low and high Ct value corresponds to the presence of higher and lower amounts of viral RNA, respectively. Usually, Ct values above 40 are treated as unspecific amplification. As clearly shown by many studies [10,14,19,25] as the pool size increases, the amplified RNA reaches the threshold later, as expected from a diluted sample. The increase in the number of Ct can be calculated with the simple formula log_(2)_ *s* = *x*, where the size of the pool *s* is used as the dilution factor and *x* is the shift in Ct. Take, for example, a positive sample that amplifies at low Ct values (Ct = 18–25). A 10-fold dilution, i.e., pooling 10 samples, would increase by about 3.3 cycles, which is well above the detection limit. While samples collected from patients with symptoms generally present high viral titers that are easy to detect [1], testing of asymptomatic patients may require extremely sensitive tests due to lower viral load. This problem is stressed for larger pooled (>100) samples [16]. Yelin et al. [25] found that a single positive sample could be detected in NA pools of 32 extracted RNA samples with a false negative rate of 10%. However, FNs can also affect smaller pools. Farfan et al. [26], for example, showed that no amplification signal was detected in a VTM pool of size 5, which included a positive sample with a Ct = 36.1. Similarly, Wacharapluesadee et al. [22] used a pool size of 10 and found a 13.3% of FNs due to positive samples with low viral load.

The dilution effect due to pooling was considered to be of varying importance among the studies, which is likely because, in the different experimental settings, samples with different viral loads were pooled. Arvind and colleagues [20] investigated the pool size in depth on the basis of different viral loads. Clinical samples previously tested as positive but with a different range of Ct values of the E gene target were chosen, serially diluted with negative sample elutes, and tested by RT-PCR to determine whether they remained detectable. The authors found that the probability of detection of the positive pool decreased as the Ct value of the single positive sample in the pool increased (lower viral load). Positive samples with Ct values from 25 to 31 were detected in pools until 1:48 dilution, while pools containing samples of Ct value 33 were detected until 1:32 dilution. Samples with Ct values of 35, 38, and 39 were detected until 1:8, 1:6, and 1:4 dilutions, respectively. For a pool size of 2 and 4, no false negative results were found, while for sizes of 6, 8, and 10, the authors calculated a negative predictive value (i.e., the probability that an individual specimen identified as negative at the end of a pooling algorithm was truly negative) that gradually decreased from 97.2% to 95.45% due to a progressive increase in false negative results.

In conclusion, a “working” pool size is also bound to the viral load of the positive samples inside it. In the most complicated scenario, we will have a pool with a single positive sample with a low viral load and, in this case, the pooling approach could lead to a false negative. Thus, the question now is: how many times can this happen in real population monitoring? This could be estimated from the calculation of the median Ct values and of the confidence interval using the data collected so far, even if these data mainly come from symptomatic patients, whereas the asymptomatic population should have a higher median Ct value. It should be useful to routinely collect the Ct value of positive samples over time and estimate the percentage of weak positive samples that are close to the assay limit of detection. Taking into account the practical limits for their own machine and kit combination, each lab may consider a smaller pool dimension to reduce the Ct shift and maintain higher sensitivity.

Additionally, increasing the cutoff in Ct for the analysis of pooled samples may increase the number of false positives (FP). However, this is not a real problem because unresolved sample from the positive pool will be sampled individually and the standard Ct cutoff can be reset to eliminate potential FP from the first round.

A ~10–15% loss in sensitivity is generally associated to a pool size pool 10–20, but this value would not likely pass the current authorization criteria by the U.S. Food and Drug Administration. Hence, at the moment, it would be useful to reach a consensus as to a suitable “conservative pool size”—a pragmatic threshold beyond which technical problems for the application of pooling could arise, lowering the sensitivity of the tests. The available data suggest a conservative size of five. Further data and a critical discussion among the researchers are, however, necessary to establish this essential information.

Unfortunately, these sizes are quite restrictive towards the potential efficiency of pooling, especially for virus frequency values below 1%. This is due to the fact that, so far, attempts have been made only to adapt pooling to the standard protocol diagnostic protocol and not vice versa. However, taking into account the dilution problems discussed above, the development of a new ad hoc protocol should also be considered.

Various proposals have already been made on this topic. The samples from NP swabs are collected into 2 mL of VTM. This volume could be reduced to 1 mL to concentrate the sample, and consequently, increase the amount of specimen that can be loaded to the extraction system. This strategy could also be useful to spare the transport medium, which is another potential bottleneck.

Different studies [22,27] suggested to increment the capability of the extraction protocol. In particular, using saliva pools, Watkins et al. [27] demonstrated that, when increasing the extraction volume (from 300 to 400 µL) and keeping elution volume constant, it was possible to improve viral detection in pooled samples to near undiluted levels.

Alternatively, the samples collected in the VTM could be concentrated by a vacuum concentrator system, but this step is time consuming. We did not find any study dedicated to the RNA recovery efficiency for the extraction step. It may be useful to verify how much elution volumes could be reduced without causing a significant drop in RNA recovery to obtain more concentrated eluates and improve sensitivity of large NA pools. Sensitivity could also be increased by raising the volume of the RT-PCR reaction to accommodate more input material to the PCR instrumentation. Otherwise, digital-droplet PCR may allow for even more sensitive testing than RT-PCR. Lu et al. reported an accuracy of 96.3% for clinical samples testing using digital PCR and were able to reveal the virus in four samples that were found to be negative by RT-PCR [28]. Furthermore, digital droplet methods have been able to detect down to 0.4 viral RNA copies/µL in patient samples [29]. Because digital PCR allows for more careful quantitation of viral RNA copy number, this highly sensitive test may also be useful to offset the drop in sensitivity due to sample pooling.

A summary of the characteristics of pooling schemes and types is shown in Table 1.

## 5. Conclusions

The unprecedented global demand for commercial kits to perform RNA extraction and RT-PCR kits due to COVID-19 emergency is leading to the establishment of new diagnostic strategies.

If the pooling protocols were applied and optimized ad hoc, we would probably no longer have the problem of laboratory reagents shortage. The bottleneck could, however, become the sticks used for the NP swabs. This risk should be timely addressed by increasing their production and availability. On the other hand, pooling can be applied to saliva, which continues to gain scientific evidences as a potential specimen to aid testing demands for SARS-CoV-2 molecular diagnosis. In fact, Ott et al., 2020 [2] clearly demonstrated that saliva can be easily self-collected in simple collection tubes without the need of sticks, expensive stabilizing buffers, or cold chain transport.

The main concern about the use of sample pooling for SARS-CoV-2 diagnosis certainly remains the risk of reducing the analysis sensitivity and of increasing the rate of false negatives. The partial loss of information due to the pooling approach should be conveniently balanced by the possibility of screening more people more often. Moreover, false negative samples generally come from subjects at the earliest stages of infection, but if the examination is repeated after a few days (rather than after a longer time), there is an excellent probability of detection, since the viral load would have increased in the meantime. This could be the case for people at high risk of infection for whom it has been calculated that there could be a significant impact in the effectiveness of covid-19 surveillance only if the monitoring frequency is at least twice a week [30]. As a matter of fact, an effective surveillance largely depends on the testing frequency and the speed of reporting, and is only marginally improved by high test sensitivity, also taking into account that, during the exponential growth of the virus, there is only a very short temporal window in which only the more sensitive test could allow a correct diagnosis [31]. Based on these observations, it has also been proposed that the high sensitivity diagnostic test based on PCR, with a limit of detection at 10^3^ copies/mL [32], should be conveniently replaced with a less sensitive but cheaper and faster test, such as the Loop Mediated Isothermal Amplification (LAMP) test [33], with a limit of detection at 10^5^ copies/mL. The same argument could be correctly applied to pooling strategies: by significantly reducing the number of analysis, pooling offers the possibility of increasing the frequency of monitoring and reducing the turnaround time. The FN issue should be seen not only from the point of view of how many FNs are expected for each single diagnostic test, but also considering which strategy allows us to minimize the number of days in which a not-correctly identified positive subject can freely circulate, putting society at risk. Finally, in this scenario, where a slightly less accurate test done repeatedly is more beneficial than an accurate test done rarely, an approach based on pooling and RT-PCR will not be affected by an increase of FP results, and this is a significant advantage over other types of rapid tests.

As could be associated with the standard protocols, reagents, and equipment, pooling could be applied immediately in current clinical testing laboratories. Moreover, this approach is compatible to automating and parallelizing the experiments as much as possible, providing a reliable procedure to identify positive subjects. All this can be done without having to develop new technologies: a different use and adaptation of the technology that is already at work as the gold standard would suffice. One last thought going back to the initial question at the beginning of this review: why is pooling still not universally adopted by clinical laboratories? Certainly, this strategy has no sponsor among the companies that develop diagnostic tests. The absence of a commercial support is also accompanied by the push towards innovative equipment, which, beyond validity, has a great economic interest.

If the hope is to find as soon as possible a reliable test for SARS-CoV-2 diagnosis that is both fast and reliable, the solution may already be at hand by applying a pooling strategy to the current standard diagnostic pipeline. We hope that this review can aid researchers in designing optimized pooling-based testing protocols to increase the availability and speed of mass scale COVID-19 testing.

## Figures and Tables

**Table 1:** A summary of the characteristics of pooling schemes and types.

Types of Pooling		
Swab pool	Obtained by adding swabs from different patients into a single volume of viral transport media.	
VTM pool	An aliquot of transport media of each sample is collected to create a homogeneous pool.	
NA pool	An aliquot of nucleic acid extracted from each sample is collected to create a homogeneous pool.	
**Schemes of Pooling**		
1D pooling and deconvolution of the positive pools: this is the simplest approach.		
Adaptative	Protocols in which the results from the earlier pools tests determine who is selected to build the subsequent pooling steps.	
	Generally include:	2D pooling: a two-step protocol in which either: (i) Positive first-level pools are split in smaller second-level subpools; or (ii) Samples are arranged on the basis of a geometrical schemes (e.g., symmetric or asymmetric matrices).
	Results	Interpretation
	A pool that gives a negative result	This pool is made up of all negative samples that can be presumed to be “true negatives” (TN).
	A pool that gives a positive result	In this pool there is at least one “true positive” (TP) sample. Samples of this pool may be labeled as “unresolved” until further testing identify the TP.
Combinatorial	Includes those protocols in which replicates of each sample are created (from 2 to 6 replicates) and multiple pool steps are performed simultaneously.	
	Results	Interpretation
	Samples with at least one replicate in a negative pool	These samples can be presumed TN.
	Samples with all its replicate in positive pools	Results obtained combining the compositions of the different pools will establish whether this sample can be considered TP or an unresolved one for which a validation test will be needed.
